# Changes in overall and regional body fatness from childhood to early adolescence

**DOI:** 10.1038/s41598-019-38486-x

**Published:** 2019-02-13

**Authors:** Leonardo Pozza Santos, Ina S. Santos, Alicia Matijasevich, Aluísio J. D. Barros

**Affiliations:** 1 Post-graduate Program in Epidemiology, Federal University of Pelotas, Pelotas, Brazil; 2 Nutrition School, Federal University of Pampa, Itaqui, Brazil; 3 Department of Preventive Medicine, Faculty of Medicine FMUSP, University of São Paulo, São Paulo, Brazil

## Abstract

Children weight gain is mostly due to fat-free mass than fat mass, but the changes in body composition dynamics related to child growth can be attributed to the obesity epidemic. We aimed to assess changes in measures of body composition from 6 to 11 years of age according to sex, and to examine whether changes in these measures are associated with sociodemographic characteristics. A longitudinal study using data from the 2004 Pelotas Birth Cohort was conducted, and assessed body composition and fat distribution through measures of BMI, fat mass index, fat-free mass index, and android and gynoid fat mass percentages from DXA. Changes in body fatness were calculated as the difference between measures collected at 6 and 11 years of age, and linear regression models were used to assess changes in body composition according to sociodemographic characteristics. An increase in mean BMI z-score from 6 to 11 years was observed only in boys and obesity prevalence reached one out of four boys and one out of five girls. There was an increase in fat mass percentage, fat mass index and android fat mass, with this effect more accentuated in boys when compared to girls. Maternal BMI was the most consistent factor associated with change in body fatness. Children from mothers with obesity showed larger increases in fat mass percentage, fat mass index and android fat mass. There was an increase in body fatness and a centralisation of body shape, mostly associated with male sex and maternal obesity. These results may indicate an early risk of non-communicable diseases in children from the Pelotas 2004 Birth Cohort.

## Introduction

Children weight gain is usually based on fat-free mass rather than fat mass, as the proportion of fat mass tends to decline during childhood^1,2^. Accentuated increases in body fat percentage is only seen after the onset of puberty, when sex differences in overall and regional body composition become more visible^1–4^. Body composition dynamics related to children’s growth, however, may be changing due to the high obesity prevalence in young populations worldwide.

In Latin America, approximately 20 million children are either overweight or obese^5^, though most investigations use body mass index (BMI) and waist circumference (WC) to assess overall obesity and central body shape. Despite being easy to apply, interpret, and compare with other studies, both BMI and WC do not provide information on fat mass and fat-free mass^6–8^. As such, this limitation does not allow us to know to what extent excessive weight gain in children is exclusively due to excessive fat mass or if there is an interactive and proportional growth of fat mass, lean mass and bone mineral content.

High proportions of total and central fat in adults is associated with disease risks and mortality^9–11^. If the body composition dynamics related to children’s growth are changing due to the childhood obesity epidemic, we can expect higher amounts of fat tissue at earlier ages. As a result, these higher concentrations of total and central fat mass earlier than the onset of puberty might further increase health risks in the short- and long- term^12,13^.

In the 2004 Pelotas Birth Cohort Study, we have information about total and regional body composition assessed when children were 6 and 11 years of age^14^. With these data, we can investigate the body composition dynamics related to children’s growth from childhood to early adolescence. Therefore, our objective was to assess the change in measures of overall and regional body composition from 6 to 11 years of age according to sex, and to examine whether changes in these measures are associated with socioeconomic and demographic characteristics.

## Methods

### Subjects

In 2004, a third birth cohort study started in Pelotas, Brazil. Pelotas is a medium-sized city (330,000 inhabitants), located around 150 km away from the Brazilian south border. Its economy is based on agriculture and commerce, and compared to the whole country, Pelotas has a lower Gross Domestic Product per capita, lower illiteracy and a higher Human Development Index.

The 2004 Pelotas Birth Cohort Study recruited 4231 new-borns in the city’s maternity hospitals, representing 99.2% of total births from mothers living in urban areas. Trained interviewers assessed mothers and their babies within 24-hours after delivery and applied a structured questionnaire containing information about the family, mother, current pregnancy, birth and child.

At the ages of 3 months, and 1, 2, 4, 6 and 11 years, the whole cohort were followed-up, and specifically trained field-workers collected information about anthropometric measures, health status, dietary intake, child development, housing conditions and socioeconomic position (SEP). Complete information about the perinatal study and all follow-up waves have been previously published^14–16^. Retention rates in all follow-ups were greater than 80%.

The Research Ethics Committee from the Medical School of Federal University of Pelotas approved all follow-ups, and the mother or legal guardian gave written informed consent to participate in the study. All methods employed in follow-ups of the 2004 Pelotas Birth Cohort Study were performed in accordance with relevant guidelines and regulations.

### Anthropometry, body composition and distribution of body fat at 6 and 11 years

When children averaged 6.8 and 10.9 years of age, they visited our research clinic centre and anthropometric and body composition assessments were performed. In anthropometric evaluation, weight was measured using a high precision scale (0.01 Kg) coupled to a BodPod machine (Cosmed, Italy, http://goo.gl/7jzfLc), while height was collected using a Harpender metal stadiometer (Holtain, Crymych, UK). We then calculated BMI by dividing weight (Kg) by height (m^2^). After, we standardized BMI using the 2007 World Health Organization (WHO) growth reference^17^, and classified children as ‘normal’ (−2 to ≤+1 s.d.), ‘overweight’ (>+1 to ≤+2 s.d.) or ‘obese’ (>+2 s.d.).

We performed body composition assessment in both follow-ups using dual-energy X-ray absorptiometry (DXA). In DXA examinations conducted at 6 and 11 years of age in our research clinic centre (Dr. Amilcar Gigante Epidemiology Research Center), children remained in supine position, barefoot and wearing light and tight-fitting clothes, with no earrings, piercings or any metallic objects. Trained field-workers conducted DXA examinations and assessed the quality of exams using the same hardware (GE Lunar Prodigy densitometer) and software (enCORE v15) in both follow-ups. The equipment was calibrated at the beginning of each working day, following the manufacturer’s recommendations.

To assess total body composition, we used information on the BMI z-score as well as the fat mass and fat-free mass index from DXA. Fat mass and fat-free mass indexes were calculated as following:

- Fat mass index = [Total fat mass (Kg)/height (m^2^)];
- Fat-free mass index = [Total fat-free mass (Kg)/height (m^2^)];

Children were classified according to their level of fat mass index using the gender-specific curves proposed by Khadilkar *et al*.^18^, in order to identify children with high (between >85^th^ and 95^th^ percentile) and very high (>95^th^ percentile) fat mass index. These cut-offs were defined to align with those used by WHO growth charts for overweight and obesity.

Due to the importance of body fat distribution to disease risks and mortality, we also used data from regional body fat measured by DXA. Android and gynoid fat mass percentage were used as indicators of central and peripheral body shape, respectively. We calculated android and gynoid fat mass percentages as following:

- Android fat mass (%) = [android fat mass (Kg)/total fat mass (Kg)] * 100;
- Gynoid fat mass (%) = [gynoid fat mass (Kg)/total fat mass (Kg)] * 100.

### Independent variables

To assess the socioeconomic and demographic information associated with changes in body fatness from 6 to 11 years of age, we used information about SEP at birth, based on National Wealth Index (IEN); an index which classifies individual’s SEP according to household goods and the household head’s education^19^. We also used information about maternal education (0–4 years, 5–8 years and 9 or more), maternal age at birth (<18, 18–35 and >35 years), maternal BMI three months after birth (normal BMI, overweight and obese), and maternal reported skin colour (white, brown and black) used here as a proxy of ethnicity.

### Statistical analyses

Means and 95% confidence intervals are presented for BMI z-scores, fat mass and fat-free mass indexes, and android and gynoid fat mass percentages. Changes in these measures from 6 to 11 years of age were calculated as the difference between measures collected at 11 years and 6 years.

We used linear regression models to check whether or not changes in measures of body fatness (BMI z-score and fat mass index) and body fat distribution (android fat mass and gynoid fat mass percentages) were associated with socioeconomic and demographic characteristics (SEP at birth, maternal education, maternal age at birth, maternal BMI and maternal reported skin colour).

In these linear regression models, the calculated difference between measures collected at 11 and 6 years was the dependent variable and the socioeconomic or demographic factor analysed was the independent variable. All analyses were adjusted for SEP at birth as this variable is well associated with all the other independent characteristics included in our study (except when SEP at birth was the independent variable).

As we tested different scenarios, we used Bonferroni corrected confidence intervals to address the multiple testing issue in the linear regression models. Variation Inflation Factor was also checked to assess for multicollinearity. All analyses were stratified by sex and performed using Stata 13.1.

## Results

We included 3135 children who were followed at 6 and 11 years of age with available information on anthropometric and DXA measures in both follow-ups. Children in the study were more likely to be boys (51.1%) and to have low SEP at birth (1^st^ or 2^nd^ quintile). More than 40% of their mothers had at least 9 years of formal education, almost 10% of mothers were adolescents at the time of birth and around 45% were classified as overweight or obese three months after birth. Most mothers reported white as their children’s skin colour (Table 1). Comparing these children with those lost to follow-up, the latter were poorer and delivered by younger mothers. They did not differ in terms of maternal education, maternal BMI and mother reported skin colour (data not shown).

**Table 1:** Socioeconomic and demographic characteristics of children followed at 6 and 11 years of age. Pelotas, Brazil (N = 3135).

Socioeconomic and demographic characteristics	Children followed at 6 and 11 years N (%)
Sex	
Boys	1601 (51.1)
Girls	1534 (48.9)
Socioeconomic position at birth (quintiles)	
1^st^ (poorest)	688 (22.0)
2^nd^	667 (21.4)
3^rd^	720 (23.1)
4^th^	523 (16.8)
5^th^(richest)	524 (16.8)
Maternal education (years)	
0–4	465 (15.0)
5–8	1290 (41.5)
≥9	1352 (43.5)
Maternal age at birth (years)	
18–35	2488 (79.4)
<18	298 (9.5)
>35	348 (11.1)
Maternal BMI (Kg/m^2^)	
Normal	1732 (56.4)
Overweight	876 (28.5)
Obese	461 (15.0)
Skin colour	
White	2084 (71.2)
Brown	453 (15.5)
Black	389 (13.3)

^*^Highest missing values for self-reported skin colour (209).

BMI - Body Mass Index.

The mean of BMI z-scores increased from +0.71 s.d. (CI 95% 0.64 to 0.79) at 6 years to +0.83 s.d. (CI95% 0.76 to 0.90) at 11 years in boys and remained stable in girls (+0.68 s.d. at 6 and +0.67 at 11 years). Regarding obesity status, while less than 20% of boys were classified as obese at 6 years, the prevalence of obesity at 11 years of age increased to 25%. In girls, obesity status increased only 1.8 percentage points, from 17.5% at 6 years to 19.3% at 11 years (Table 2).

**Table 2:** Mean of overall and regional body fatness, and prevalence of overweight and obesity at 6 and 11 years of age, stratified by sex. Pelotas, Brazil (N = 3135).

Measures of total and regional fat mass^a^	6 years	11 years
	Mean (sd)	Mean (sd)
*BMI (z-score)*	*0*.*484*	*0*.*002*
Male	0.72 (1.5)	0.83 (1.4)
Female	0.68 (1.4)	0.67 (1.4)
*Fat mass index (Kg/m*^*2*^)	<*0*.*001*	<*0*.*001*
Male	3.26 (2.3)	5.54 (3.5)
Female	4.25 (2.5)	6.29 (3.4)
*Fat-free mass index (Kg/m*^2^)	<*0*.*001*	<*0*.*001*
Male	13.6 (0.9)	14.2 (1.3)
Female	12.8 (0.9)	13.6 (1.4)
*Android fat mass (%)*	<*0*.*001*	*0*.*059*
Male	6.65 (1.4)	7.24 (1.7)
Female	7.18 (1.5)	7.35 (1.6)
*Gynoid fat mass (%)*	<*0*.*001*	*0*.*011*
Male	23.83 (3.5)	21.37 (3.1)
Female	22.46 (3.4)	21.09 (2.9)
**BMI status** ^**b**^	**6 years**	**11 years**
	**N (%)**	**N (%)**
*p-value*	*0*.*485*	<*0*.*001*
*Boys*		
Normal	1027 (64.7)	853 (53.3)
Overweight	283 (17.8)	348 (21.7)
Obese	277 (17.5)	400 (25.0)
*Girls*		
Normal	959 (63.0)	876 (57.1)
Overweight	296 (19.5)	365 (23.8)
Obese	267 (17.5)	293 (19.1)
**Fat mass index status** ^**b**^	**6 years**	**11 years**
	**N (%)**	**N (%)**
*p-value*	*0*.*634*	<*0*.*001*
*Boys*		
< = 85^th^ Percentile	1044 (66.3)	854 (54.3)
>85^th^–95^th^ Percentile	240 (15.3)	415 (26.4)
>95^th^ Percentile	290 (18.4)	305 (19.4)
*Girls*		
< = 85^th^ Percentile	999 (66.2)	975 (64.6)
>85^th^–95^th^ Percentile	216 (14.3)	228 (15.1)
>95^th^ Percentile	294 (19.5)	306 (20.3)

^a^displayed p-value from Analysis of Variance for the difference between boys and girls.

^b^displayed p-value from Pearson’s chi-squared test for the difference between boys and girls.

BMI - Body Mass Index.

Fat mass and fat-free mass indexes increased in both boys and girls from 6 to 11 years, however the increase in fat mass index was more accentuated; from 4.2 to 6.3 Kg/m^2^ in girls and from 3.3 to 5.5 Kg/m^2^ in boys. At both time-points, girls presented a higher fat mass index while boys presented a higher fat-free mass index (p-value < 0.001) (Table 2). When we assessed fat mass index classification according to Khadilkar *et al*. (2013) percentiles, we observed similar results to BMI status. There was no difference in fat mass index classification according to gender at 6 years then at 11 years of age, the proportion of children being above the 85^th^ percentile was higher in boys (45% vs. 35%) (Table 2).

Regarding body fat distribution, android fat mass percentage increased from 7.2% (CI95% 7.1 to 7.3) to 7.4% (CI 95% 7.3 to 7.4) in girls, and from 6.7% (CI 95% 6.6 to 6.7) to 7.2% (CI 95% 7.2 to 7.3) in boys (Table 2). On the other hand, the proportion of gynoid fat mass decreased from 6 to 11 years in both sexes. Girls presented 22.5% (CI 95% 22.3 to 22.6) of gynoid fat mass at 6 years and 21.1% (CI 95% 20.9 to 21.2) at 11 years; boys presented 23.8% (CI95% 23.7 to 24.0) of gynoid fat mass at 6 years and 21.4% (CI 95% 21.2 to 21.5) at 11 years (Table 2).

Table 3 shows changes in BMI z-scores and fat mass indexes from 6 to 11 years according to the independent variables included in our study. Compared to the less-affluent children, boys and girls with higher SEP at birth presented smaller increases in BMI z-score from 6 to 11 years. Children from overweight and obese mothers presented larger increases in fat mass index, independent of SEP at birth. Finally, black girls presented a larger increase in BMI z-score from 6 to 11 years of age when compared to the white girls (Table 3).

**Table 3:** Changes in BMI z-score and fat mass index from 6 to 11 years according to socioeconomic and demographic characteristics, stratified by sex. Pelotas, Brazil (N = 3135).

	BMI z-score		Fat mass index (Kg/m^2^)	
	Boys β (95% CI)*	Girls β (95% CI)*	Boys β (95% CI)*	Girls β (95% CI)*
SEP at birth (quintiles)	*0*.*003*	*0*.*001*	*0*.*556*	*0*.*222*
1^st^ quintile	0.00	0.00	0.00	0.00
2^nd^ quintile	−0.03 (−0.20; 0.14)	−0.03 (−0.17; 0.11)	0.13 (−0.27; 0.54)	−0.07 (−0.42; 0.28)
3^rd^ quintile	−0.06 (−0.23; 0.10)	−0.06 (−0.20; 0.07)	0.24 (−0.15; 0.63)	0.03 (−0.32; 0.38)
4^th^ quintile	−0.15 (−0.32; 0.03)	**−0.23 (−0.39; −0.08)**	0.21 (−0.21; 0.63)	−0.29 (−0.67; 0.10)
5^th^ quintile	**−0.25 (−0.43; −0.07)**	−0.13 (−0.28; 0.02)	0.22 (−0.20; 0.64)	−0.17 (−0.56; 0.21)
Maternal education (years)^†^	*0*.*131*	*0*.*066*	*0*.*576*	*0*.*273*
0–4	0.00	0.00	0.00	0.00
5–8	−0.10 (−0.26; 0.05)	−0.04 (−0.17; 0.09)	−0.13 (−0.50; 0.23)	0.02 (−0.30; 0.34)
≥9	−0.15 (−0.32; 0.02)	−0.13 (−0.26; 0.01)	−0.02 (−0.42; 0.37)	−0.16 (−0.50; 0.19)
Maternal age at birth (years)^†^	*0*.*474*	*0*.*239*	*0*.*395*	*0*.*022*
18–35	0.00	0.00	0.00	0.00
<18	0.08 (−0.09; 0.25)	−0.08 (−0.23; 0.06)	−0.04 (−0.44; 0.35)	−0.42 (−0.78; −0.05)
>35	0.05 (−0.11; 0.21)	−0.07 (−0.21; 0.06)	0.22 (−0.16; 0.61)	−0.20 (−0.54; 0.13)
Maternal BMI three months after birth^†^	*0*.*079*	*0*.*002*	<*0*.*001*	<*0*.*001*
Normal	0.00	0.00	0.00	0.00
Overweight	0.12 (0.00; 0.23)	**0.14 (0.04; 0.24)**	**0.62 (0.36; 0.89)**	**0.75 (0.51; 1.00)**
Obese	0.03 (−0.11; 0.18)	**0.13 (0.01; 0.25)**	**1.06 (0.72; 1.41)**	**1.07 (0.77; 1.37)**
Skin colour^†^	*0*.*625*	*0*.*006*	*0*.*093*	*0*.*524*
White	0.00	0.00	0.00	0.00
Brown	−0.04 (−0.19; 0.10)	0.06 (−0.07; 0.18)	−0.12 (−0.47; 0.22)	−0.06 (−0.37; 0.26)
Black	−0.06 (−0.21; 0.10)	**0.19 (0.06; 0.33)**	−0.35 (−0.71; 0.01)	0.15 (−0.20; 0.49)

*Linear regression using Bonferroni correction; ^†^Adjusted for SEP at birth; BMI - Body Mass Index; SEP - Socioeconomic Position.

Table 4 shows changes in android and gynoid fat mass percentage from 6 to 11 years according to the same co-variables. Boys and girls from overweight and obese mothers presented larger increases in android fat mass percentage, independent of SEP at birth. Android and gynoid fat mass percentage from 6 to 11 years did not differ at all according to the other factors included in this analysis (Table 4).

**Table 4:** Changes in proportion of android and gynoid fat mass from 6 to 11 years according to socioeconomic and demographic characteristics, stratified by sex. Pelotas, Brazil (N = 3135).

	Android fat mass (%)		Gynoid fat mass (%)	
	Boys β (95% CI)*	Girls β (95% CI)*	Boys β (95% CI)*	Girls β (95% CI)*
SEP at birth (quintiles)	*0*.*689*	*0*.*137*	*0*.*022*	*0*.*016*
1^st^ quintile	0.00	0.00	0.00	0.00
2^nd^ quintile	0.08 (−0.14; 0.31)	−0.12 (−0.32; 0.07)	−0.12 (−0.58; 0.34)	0.11 (−0.30; 0.53)
3^rd^ quintile	0.09 (−0.13; 0.31)	−0.06 (−0.25; 0.13)	0.23 (−0.22; 0.67)	0.26 (−0.15; 0.67)
4^th^ quintile	0.11 (−0.13; 0.34)	−0.21 (−0.43; 0.00)	0.22 (−0.26; 0.70)	0.51 (0.06; 0.96)
5^th^ quintile	0.13 (−0.11; 0.36)	−0.07 (−0.28; 0.14)	0.48 (0.00; 0.95)	0.47 (0.02; 0.92)
Maternal education (years)^†^	*0*.*525*	*0*.*736*	*0*.*136*	*0*.*402*
0–4	0.00	0.00	0.00	0.00
5–8	0.01 (−0.19; 0.22)	0.05 (−0.13; 0.23)	0.27 (−0.14; 0.69)	0.16 (−0.21; 0.54)
≥9	−0.06 (−0.29; 0.16)	0.01 (−0.18; 0.20)	0.40 (−0.05; 0.85)	0.24 (−0.16; 0.64)
Maternal age at birth (years)^†^	*0*.*159*	*0*.*072*	*0*.*057*	*0*.*164*
18–35	0.00	0.00	0.00	0.00
<18	−0.01 (−0.23; 0.22)	−0.21 (−0.41; 0.00)	−0.43 (−0.88; 0.02)	0.32 (−0.11; 0.76)
>35	0.18 (−0.03; 0.40)	0.00 (−0.16; 0.17)	−0.26 (−0.70; 0.18)	−0.12 (−0.51; 0.27)
Maternal BMI^†^	<*0*.*001*	*0*.*011*	*0*.*344*	*0*.*545*
Normal	0.00	0.00	0.00	0.00
Overweight	**0.16 (0.01; 0.32)**	**0.15 (0.01; 0.29)**	−0.20 (−0.51; 0.12)	−0.03 (−0.33; 0.26)
Obese	**0.33 (0.14; 0.53)**	**0.18 (0.01; 0.35)**	−0.13 (−0.53; 0.27)	0.15 (−0.21; 0.52)
Skin colour^†^	*0*.*075*	*0*.*978*	*0*.*169*	*0*.*122*
White	0.00	0.00	0.00	0.00
Brown	−0.17 (−0.36; 0.03)	−0.02 (−0.19; 0.16)	0.25 (−0.15; 0.65)	0.10 (−0.27; 0.47)
Black	−0.14 (−0.35; 0.06)	−0.01 (−0.19; 0.18)	0.28 (−0.13; 0.70)	−0.32 (−0.72; 0.08)

*Linear regression using Bonferroni correction; ^†^Adjusted for SEP at birth; BMI - Body Mass Index; SEP - Socioeconomic Position.

## Discussion

Our study showed changes in overall and regional body fatness from 6 to 11 years of age in a population-based sample from Pelotas, Brazil. At 6 years of age, there was no difference in BMI z-scores according to sex, however when children were 11 years old, BMI z-scores became higher in boys. In addition, one out of four boys and one out of five girls were classified as obese when they were 11 years old.

Recent estimates from Brazil and other settings have already shown an increase in obesity prevalence in all age groups^20–22^. In Brazil as a whole, the last national representative survey showed a prevalence of obesity in 5 to 9 year-old children of 17% and 12% in boys and girls, respectively^23^. Despite the prevalence of obesity having increased in Brazilian children, the proportion of obesity in children from the 2004 Pelotas Birth Cohort is higher, mainly in boys, reaching one quarter of the sample. Furthermore, a notable number of children from our cohort were classified with high fat mass indexes, even when referenced against Indian children who are known to have higher amounts of body fatness than Western children^18^.

Children included in our study had not started the onset of puberty or, at best, were in the early stages of it. Due to this reason, we might expect that gain in total body mass would be mostly due to increases in fat-free mass than fat mass, as before the onset of puberty, weight gain is supposed to be based on fat-free mass rather than fat mass^1^. We observed, however, that the fat mass index increased in the whole cohort, independent of socioeconomic and demographic characteristics.

The most consistent factor associated with increases in body fatness from 6 to 11 years was maternal BMI. These results are consistent with other reports of maternal BMI and offspring’s body composition in this cohort and other studies^24–26^. An investigation using data from the 2004 Pelotas cohort study showed a positive association between pre-pregnancy BMI and offspring body composition at 6 years of age as measured by air-displacement plethysmography^24^. Furthermore, Pacce *et al*.^26^ and Zalbahar *et al*.^25^ also found a positive relationship between maternal BMI and offspring’s body composition. Despite cross-sectional association between pre-pregnancy maternal BMI and offspring’s body composition having been well reported in the literature, our study went further and showed that children from mothers with obesity presented larger increases in BMI z-scores, fat mass indexes and android fat mass percentages from late childhood to early adolescence.

But what would be the reasons involved in high prevalence of obesity and higher storage of fat mass from childhood to early adolescence? Unhealthy feeding habits may be one aspect related to this process. Recently published research using data from this cohort showed that more than 40% of daily energy intake at 6 years came from ultra-processed foods^27^. It is an interesting finding as other studies have shown that ultra-processed foods are associated with a higher risk of obesity^28,29^. Additionally, as physical activity levels decrease considerably from childhood to adolescence^30–32^, sedentarism might be another factor associated with body fatness increases in this cohort. Nevertheless, we did not address this association in our study and further investigations would be interesting to test this hypothesis.

Our sample also presented a centralisation of body fatness from 6 to 11 years, despite the modest increase in measures of android fat mass (0.5 percentage point in boys and 0.2 percentage point in girls). Hoffman *et al*.^33^, using data from a Brazilian cohort, showed an increase in trunk fat mass percentage in children entering puberty, indicating a centralisation of fatness. Nonetheless, despite fat mass centralisation observed in our cohort, the decrease in peripheral body fat was a little bit more accentuated, reaching more than two percentage points in boys. This may be associated with the prevalence of obesity, but may also be part of the maturing process faced by these children. Independent of the reasons for body fat centralisation, higher amounts of central body fat is associated with disease risks and mortality, and an early exposure to android body shape can increase these risks even more^10,12,34^.

Large sample size and low attrition rates at both 6-year and 11-year follow-ups can be considered a strength of our study. A further strength is the comprehensive body composition assessment at both time-points using DXA, which allowed us to look at the evolution of children’s body fatness from late childhood to early adolescence beyond BMI.

Descriptive analyses with no adjustment for potential confounders can be treated as a limitation of our study, since associations observed here may be confounded by other factors. In addition, the unavailability of pubertal stage information for boys meant we were unable to address the potential role of pubertal stage on the evolution of body fatness/body fat distribution in this study. Finally, the use of multiple analyses in our study may be considered another limitation, increasing the probability of type-I error. Nevertheless, the use of Bonferroni’s correction and the good power of our sample helped in addressing this matter.

In conclusion, there was an increase in overall body fatness as well as a centralisation of body shape from late childhood to early adolescence, associated with male sex and maternal obesity soon after the delivery. These results indicate that children from the 2004 Pelotas Birth Cohort Study may be at a higher risk of non-communicable diseases in the mid- and long-term, and actions to address this problem are needed.

## Data Availability

Due to confidentiality conditions, the authors were only allowed to publish analytic results from the data, but not the data itself.
